# Colour preference and foraging constancy in the Asian giant honeybee *Apis dorsata*

**DOI:** 10.1242/jeb.252021

**Published:** 2026-06-04

**Authors:** Sudeep R, Sachin Bhaskar, Hema Somanathan

**Affiliations:** School of Biology, Indian Institute of Science Education and Research Thiruvananthapuram, Thiruvananthapuram 695551, Kerala, India

**Keywords:** Floral constancy, Reward expectation, Spontaneous preference

## Abstract

Tropical pollinators forage in spatiotemporally variable environments requiring flexible strategies that remain understudied in the tropics. Floral constancy – the temporary restriction to a single flower type – influences foraging success and plant–pollinator interactions.

We examined colour preferences and constancy in wild colonies of the Asian giant honeybee, *Apis dorsata*, testing how reward concentration and learning shape these behaviours and whether the spectral composition of the floral community correlates with bee colour preferences. Using blue–yellow dimorphic artificial flower arrays, we tested whether: (1) reward quality modulates spontaneous colour preference and constancy, with low reward promoting reliance on spontaneous biases and high reward promoting exploratory choices; (2) recent experience modifies these biases; and (3) the floral community colour composition corresponds with bee preferences. Bees trained to a neutral UV–grey stimulus were expected to revert to their spontaneously preferred colour. Bees showed strong preference for and high constancy to blue. This short-wavelength bias was strongest under low reward and weakened at high reward concentrations, demonstrating reward-dependent shifts in behaviour. We propose reward concentration modulates arousal, with low reward favouring spontaneous preferences and high reward promoting exploratory sampling. Bees readily learned to prefer yellow with training and sequential exposure to both colours eliminated overall preference, indicating that recent experience disrupts constancy. The community was dominated by short-wavelength flowers, consistent with possible pollinator-mediated selection linked to the blue preference in *A. dorsata.* Overall, colour preferences in *A. dorsata* are flexible, shaped by reward quality and recent experience, with key consequences for floral constancy and plant–pollinator interactions.

## Teaser

**Summary:** Reward concentrations drive spontaneous preference and floral constancy in the tropical honeybee *Apis dorsata*, highlighting the importance of species-specific behavioural studies.

## INTRODUCTION

The paleotropical regions of Asia are underrepresented in studies on pollinator behaviour, sensory ecology and plant–pollinator interactions. Unsurprisingly, the foraging ecology of tropical Asian honeybee species remains poorly studied, with most work focusing on the eastern honeybee *Apis cerana* (Wells et al., 1992; Wells and Rathore, 1994), which is facilitated by its domestication for apiculture (Somanathan, 2024). Although the visual ecology of the western honeybee *Apis mellifera* is well understood, Asian honeybee species exhibit substantial variation, highlighting the need to better understand visual behaviour in tropical pollinators (Grüter and Ratnieks, 2011; Somanathan, 2024; Somanathan and Balamurali, 2023). Such species-level differences probably reflect the distinct ecological contexts in which tropical pollinators have evolved. Tropical habitats are characterised by high richness of plants and pollinators (Ollerton et al., 2006; Somanathan, 2024; Vizentin-Bugoni et al., 2018), greater floral phenological diversity and a wider range of plant reproductive strategies compared with those reported from temperate regions (Morellato et al., 2013; Newstrom et al., 1994). It is therefore important to examine the foraging behaviour of tropical bees in their native habitats. Our study species, the Asian giant honeybee *Apis dorsata*, is a facultatively nocturnal forager with anatomical adaptations for dim-light conditions, including nocturnal colour vision (Somanathan et al., 2009; Vijayan et al., 2023). This makes *A. dorsata* a particularly interesting species for understanding the sensory mechanisms underlying foraging behaviour.

Foraging pollinators encounter a wide variety of flowers while searching for resources, yet many display temporary fidelity to a single flower species during a foraging bout while avoiding other co-occurring flowers (Amaya-Márquez, 2009; Chittka et al., 1999; Grant, 1950; Grüter and Ratnieks, 2011; Waser, 1986). This behaviour, known as floral constancy, has been documented in diverse pollinator groups, including bees (Grant, 1950; Shrotri et al., 2024 preprint; Slaa et al., 1998; Somanathan et al., 2019; Thomson, 1981; Waser, 1986; Wells and Rathore, 1994), birds (Schmid et al., 2016), butterflies (Goulson and Cory, 1993; Lewis, 1986; Weiss, 2001) and flies (Goulson and Wright, 1998; Weiss, 2001). Floral constancy has important consequences for plant fitness by increasing the probability of conspecific pollen transfer. The adaptive value of constancy in pollinators has been discussed in relation to neurosensory constraints in recalling diverse floral traits simultaneously (Gegear and Laverty, 2005; Goulson, 2000; Goulson et al., 1997; Hill et al., 2001; Ishii, 2005; Ishii and Masuda, 2014). Such constraints are thought to be alleviated by specialising on one floral type, thereby enhancing search efficiency and reducing handling time, which leads to the effective utilisation of rewards (Amaya-Márquez, 2009; Chittka et al., 1999; Grüter et al., 2011; Grüter and Ratnieks, 2011; Hill et al., 2001; Takagi and Ohashi, 2025). Pollinators can show varying degrees of floral constancy depending on factors such as colour, odour, reward quality and quantity, and the spatial distribution, size and abundance of flowers (Baude et al., 2011; Bruninga-Socolar et al., 2022; Gegear and Laverty, 2004, 2005; Hempel de Ibarra et al., 2001; Hill et al., 1997; Ishii and Kadoya, 2016; Nityananda and Chittka, 2021; Slaa et al., 2003; Spaethe et al., 2001, 2014).

Colour is important for visually guided pollinators such as bees, many of which show innate biases for particular floral spectral wavelengths (Chittka and Menzel, 1992; van der Kooi et al., 2019). For example, flower-naïve individuals of the western honeybee *A. mellifera,* the temperate bumblebee *Bombus terrestris* and Indian stingless bee *Tetragonula iridipennis* prefer short-wavelength colours such as UV–blue, blue and violet, whereas the eastern honeybee *A. cerana* is biased toward longer wavelengths such as blue-green and lime-yellow (Balamurali et al., 2018; Giurfa et al., 1995; Gumbert, 2000). These studies demonstrate interspecific variation in innate colour preferences among bees. Innate colour preferences may provide a template for a ‘search image’, representing coevolutionary interactions between floral signals and the sensory and neural capabilities of pollinators (Menzel, 1985). Although testing hardwired innate bias requires colour-naïve individuals, experimental manipulations may influence brain development and behaviour, making it unclear whether test choices accurately reflect innate preferences in natural contexts even in species where naïve individuals can be tested (Balamurali et al., 2018). This limitation is amplified in our study species *A. dorsata*, where colonies are wild and prior experience is unknown. However, previous work demonstrates that training to a neutral stimulus such as UV–grey can erase learned colour associations and induce a reversion to innate preferences in *A. cerana* and *B. terrestris* (Balamurali et al., 2018; Gumbert, 2000). Following this approach, in this study we trained *A. dorsata* individuals to a neutral UV–grey stimulus and expect that this procedure resets any acquired colour preferences. We refer to the subsequent colour choices of these individuals as spontaneous rather than innate.

Innate or spontaneous colour preferences in pollinators are highly plastic, with individuals rapidly learning and modifying their choices based on the local floral environment and prior experience (Chittka and Raine, 2006; Dyer and Chittka, 2004; Giurfa et al., 1995; Kinoshita et al., 2017; Menzel and Shmida, 1993; Raine and Chittka, 2007b). Experience with alternative rewarding floral colours can override these biases (Giurfa et al., 1995; Heinrich et al., 1977; Hempel de Ibarra et al., 2014; Menzel, 1985), facilitating the exploration of a broader range of resources in dynamic floral environments. How innate and learned preferences interact neurologically remains debated; one view proposes that early colour learning rapidly replaces innate biases (Giurfa et al., 1995; Heinrich et al., 1977; Menzel, 1985), whereas another suggests that these biases persist throughout a forager's lifetime and continue to influence decisions in unfamiliar contexts (Brito et al., 2015; Gumbert, 2000; Lunau and Maier, 1995). Together, these perspectives indicate a close relationship between innate preferences and the formation and recall of colour memories, although the underlying mechanisms remain unresolved.

Despite extensive research on the interplay between such colour biases, learning and motivational state in temperate bees, including bumblebees and *A. mellifera* (Gumbert, 2000; Lajad and Arenas, 2025), these processes remain poorly understood in tropical bee species that forage in highly diverse and variable floral environments. How these factors interact to shape floral constancy under natural foraging conditions has received limited attention, and the role of reward attractiveness in modulating both spontaneous preferences and constancy remain unexplored in the popularly studied *A. mellifera* and *B. terrestris*.

Floral constancy has been examined using a range of approaches to understand this behaviour across ecological contexts. These include field observations that track pollinator movement (Bruninga-Socolar et al., 2022; De Jager et al., 2011; Raine and Chittka, 2007a; van der Niet et al., 2020; Waser, 1986; Wilson and Stine, 1996), analyses of pollen loads on pollinator bodies (Raine and Chittka, 2005b; Shrotri et al., 2024 preprint; Somanathan et al., 2020; Yourstone et al., 2023), tracking movement using quantum dots (Minnaar and Anderson, 2019), laboratory experiments using artificial flower arrays (Grüter et al., 2011; Heinrich et al., 1977; Ishii and Masuda, 2014; Takagi and Ohashi, 2025; Wells et al., 1992; Wells and Rathore, 1994) and more recently, agent-based modelling (Hayes and Grüter, 2023).

Understanding behaviours such as constancy, spontaneous preferences and learning is essential for interpreting broader evolutionary and community-level associations between pollinators and floral signals. Colour biases are thought to arise from long-term evolutionary interactions between pollinators and flowers. Because insect colour vision predates the radiation of angiosperms, floral colours are likely to have evolved under pollinator-mediated selection (Chittka and Thomson, 2001). Several studies have examined correspondence between dominant floral spectra and pollinator colour biases at the community level (Ellis et al., 2021; Ishii et al., 2019; Kemp et al., 2019; Reverté et al., 2016; Shrestha et al., 2019). However, such associations remain largely unexplored in the Asian tropics.

To address these gaps in a tropical context, we focus on a wild, free-foraging honeybee species. Here, we investigate floral constancy to colour in naturally occurring colonies of the Asian giant honeybee *Apis dorsata*, a common open-nesting species found throughout south and southeast Asia (Vijayan and Somanathan, 2023). Unlike the cavity-nesting *A. mellifera* and *A. cerana*, *A. dorsata* cannot be maintained in managed hives, and experiments were conducted on foragers recruited from wild colonies. Consequently, prior foraging experience is unknown, and completely naïve individuals cannot be obtained.

Using blue and yellow dimorphic artificial flower arrays provisioned with sugar solution as reward, we tested the hypothesis that spontaneous colour preference and constancy is modulated by the quality (concentration) of the sugar reward experienced during training and testing. We predicted that bees trained to low concentration sucrose solution associated with a UV–grey neutral stimulus would rely on their spontaneously preferred colour during tests, as training to a neutral UV–grey stimulus is known to reset their previously formed floral colour–reward association in other bees. As stated earlier, we reiterate here that since *A. dorsata* colonies are wild not amenable to domestication, the experience of foragers is unknown. However, as reported in sympatric *A. cerana* (Balamurali et al., 2018) and temperate *B. terrestris* (Gumbert, 2000), we expected that UV–grey training would erase prior experience and cause a reversal to the spontaneously preferred colour in this species also; and that bees trained to high concentration reward would become exploratory in their colour preferences probably as a result of their higher arousal state.

Next, we hypothesised that colour preference and constancy would be modulated by recent colour experience. We predicted that bees trained on the less preferred colour (known from the previous experiments would show a learnt preference for this colour and that bees trained to an alternative sequence of blue and yellow stimuli would show a preference for the last trained colour.

Finally, a prevalent hypothesis suggests that an innate or spontaneous colour preference for blue exists in bees (Giurfa et al., 1995; Gumbert, 2000) which may align with the dominant floral colour in plant communities (Dyer et al., 2021; Gumbert et al., 1999; Lunau and Maier, 1995). Therefore, we asked whether the spectral properties of the surrounding floral community (a 2 km foraging range around colonies) correlated with the spontaneous colour preference in *A. dorsata* after training to a neutral UV–grey stimulus.

## MATERIALS AND METHODS

### Study species and site

**Fig. 1.**
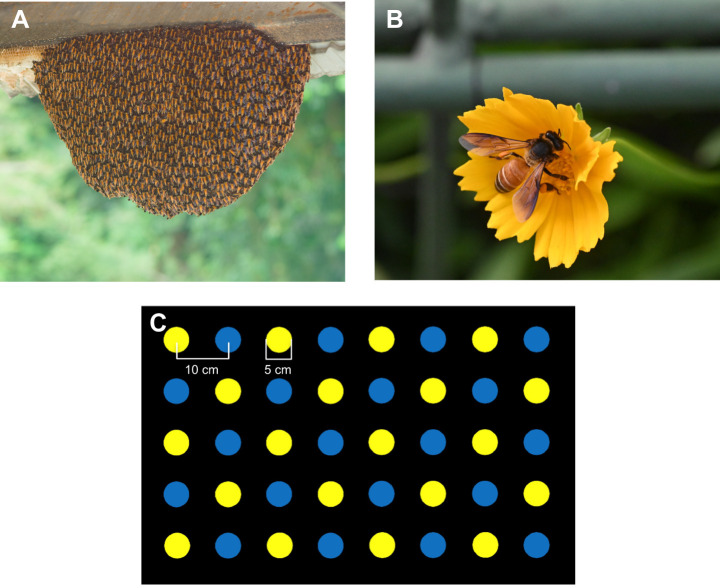
**Study organism and test array.** (A) An *Apis dorsata* colony on the roof overhang of the Biological Sciences Building, IISER Thiruvananthapuram. (B) An individual *A. dorsata* bee on a flower (*Cosmos sulphureus*). (C) Schematic of the layout of the blue and yellow test array.

We conducted our experiments using foragers from wild *Apis dorsata* Fabricius 1793 colonies nesting on the roof overhangs and window ledges of the Biological Science Building at the Indian Institute of Science Education and Research Thiruvananthapuram (IISER TVM; 08°40′55.5398″ N, 077°08′08.4215″ W) (Fig. 1A, Fig. S1). During the study period from December 2020 to August 2022, colonies underwent episodes of swarming and migration (Vijayan and Somanathan, 2024), so the number of active colonies fluctuated from a maximum of 16 to a minimum of three*.*

Foragers were first recruited to visit a black feeder placed in the open and containing sugar solution. After a sufficient number of bees had been recruited, the black feeder was gradually moved in incremental steps into an experimental arena which was located on the roof terrace about 5–10 m from the hives. The arena consisted of an aluminium cage (1 m×1 m×0.75 m) with nylon mesh sides and roof. A video camera (GoPro Hero 7 Black) was mounted at the top of the cage to record the experiments. Bees entered the arena through a clear Plexiglass tunnel equipped with shutters to control the number of bees entering the arena (Fig. S2).

### Stimuli

Matte finish vinyl stickers (LG Hausys) pasted on clear 5 cm diameter acrylic discs were used as training and test stimuli in the experiments. UV–grey stimuli made from matte finish UV reflecting cardboard sheets were pasted on the clear acrylic discs. Matte black vinyl stickers were used as the background on which the stimuli were presented in all the experiments described below. The spectral reflectance of the stimuli was measured using a spectrophotometer (Ocean Optics Ocean-HDX UV-vis spectrometer, supplemented by a PX-2 pulsed Xenon light source; Fig. S3). The colour loci of the stimuli were modelled in the hexagonal colour space (Chittka, 1992) using the *pavo* package (https://CRAN.R-project.org/package=pavo; Maia et al., 2013) in R version 4.5.1 (r-project.org), to ensure that bees could discriminate the stimuli from the background both achromatically and chromatically (Table T1).

Experiments commenced after an adequate number of bees learnt to enter the arena and consisted of three phases: a pre-training phase, a training phase and a test phase. The pre-training phase was identical in all experiments: bees that were recruited into the arena were presented with a pair of clear acrylic discs at the centre of which sugar solution was provided *ad libitum*. While they were feeding, bees were individually marked on the thorax and abdomen using combinations of acrylic paint colours (Camel Fabrica, Camlin Kokuyo, India) to identify individuals. Following this, marked individuals were allowed to enter the arena and forage from a different set of four clear acrylic discs placed on the arena floor for three consecutive foraging bouts. The positions of the discs were changed frequently to encourage bees to explore all the discs and to avoid any positional bias. Only bees that made two or more flight landings were used for analysis and crawling/walking behaviours were discarded. Sample sizes for all experiments ranged from 19 to 22, as commonly used in similar studies (Grüter et al., 2011; Takagi and Ohashi, 2025; Wells and Rathore, 1994). Variables quantified in our experiments include the following. (a) Bout, which is defined as all the stimuli choices made by a bee from the time of entering to exiting the arena. (b) First choice, which refers to the first colour stimulus a bee landed on during a test. (c) Total choices, which reflects the total number of stimuli of a particular colour that a bee landed on during a bout. All bees were euthanised after they completed tests, by capturing them in a collection vial containing 90% ethanol.

### Spontaneous constancy to colour under different training and test reward concentrations

To examine spontaneous constancy and whether it is modulated by reward quality (sugar concentration), we performed three experiments across which the reward concentration differed during the training and test phases. In experiments 1–3, we followed a common protocol where marked bees were trained to the arena and allowed to feed on 30 μl sugar solution at varying concentrations provided on four UV–grey stimuli. Based on previous studies, the UV–grey stimulus serves as a neutral achromatic stimulus that erases learned associations and prompts bees to rely on their spontaneously preferred colour while making foraging decisions (Balamurali et al., 2018; Gumbert, 2000). We assumed that this is also the case in *A. dorsata* as we had no knowledge of the prior experience of bees in these wild colonies. The stimuli positions were changed during training to encourage exploration and avoid positional learning. Each UV–grey stimulus contained 30 μl sugar solution and was replenished after a bee moved to feed from another stimulus. A training bout was considered successful if the marked bee fed from multiple stimuli before leaving the arena. Since the bees fed from three to four stimuli during each training bout and each training stimulus contained 30 μl of sugar solution, the average crop capacity of bees was estimated to be between 90 μl and 120 μl. After the completion of four training bouts, bees were tested on their next return to the arena. In tests, bees entering the arena encountered an alternative array of 20 blue and 20 yellow stimuli arranged in a grid consisting of five rows and eight columns. The distance between any two stimuli (from centre to centre) was 10 cm (Fig. 1C). Each stimulus was loaded with 5 μl sugar solution. Providing a small volume of sugar solution encouraged bees to visit multiple stimuli during a test before they reached satiation and exited the arena. To minimise the possibility that bees visited other foraging resources in the habitat, we included only marked individuals that completed training and returned for the test within 2 min. Only a single bee was allowed to enter the arena at a given time during training and testing to avoid social learning. Landing on a stimulus was recorded as a choice in tests. Tested bees were euthanised to avoid pseudoreplication. Tests were recorded (1920×1080 pixel resolution at 30 frames s^−1^) using a video camera (GoPro Hero 7 Black) and were later manually analysed frame-by-frame using QuickTime Player (version 10.5). The three experimental reward conditions that the bees experienced are described below. We used different cohorts of bees for each of the experiments as it was difficult to use the same bees in all experiments.

#### Experiment 1: training and testing with low reward concentration (both at 10%)

This experiment was designed to test for constancy to colour under low-reward conditions, after training to a neutral colour stimulus. Training to neutral colour is known to induce a return to spontaneous colour preferences in previous studies on other bee species (Balamurali et al., 2018; Gumbert, 2000). We first trained bees to feed from four UV–grey stimuli, each provisioned with 10% sugar solution (w/w). After completing four bouts of training, each bee was tested in the blue–yellow array where all stimuli contained 10% sugar solution (*n*=22, Fig. 1C).

#### Experiment 2: training with high reward concentration (30%) and testing with low reward concentration (10%)

In this experiment, we tested whether training to a higher concentration reward alters the colour preference in tests when the bees are presented with low-concentration sugar reward. During training, the UV–grey stimuli were provisioned with 30% sugar solution (*n*=21). Each bee completed four training bouts with the UV–grey stimulus before it was tested (as mentioned above) using the blue–yellow stimuli containing 10% sugar solution.

#### Experiment 3: training and testing with high reward concentration (both at 30%)

In this experiment, we examined colour choices of bees when both training and tests were carried out under consistently high-reward conditions. Bees were first trained on a neutral UV–grey stimulus as in the previous two experiments, each provisioned with 30% sugar solution. After the completion of four training bouts, each bee was tested using the blue and yellow array, each of which contained 30% sugar solution (*n*=19).

### Effect of recent foraging experience on constancy

To examine if the spontaneous constancy to blue colour that was observed in the above experiments was affected by recent foraging experience, we performed two experiments in which bees received four bouts of training to either the less preferred yellow or alternatively on blue and yellow.

#### Experiment 4: training to yellow

A set of bees received four bouts of training on the less preferred yellow (*n*=22) containing 30 μl of 10% sugar solution and then tested with the blue–yellow stimulus array each of which was rewarded with 5 μl of 10% sugar solution.

#### Experiment 5: training to alternate blue and yellow sequences

Next, we addressed the effect of recent foraging experience on colour preference and constancy. Bees were trained in an alternating sequence of bouts to both blue and yellow stimuli provided with 30 μl of 10% sugar solution. All bees were trained on each colour alternately; in one set of bees, the first training bout was to blue and the last training bout was to yellow (blue–yellow–blue–yellow–blue–yellow–blue–yellow, *n*=20) and another set of bees was trained in the opposite sequence (yellow–blue–yellow–blue–yellow–blue–yellow–blue, *n*=20). These bees were tested on an array where both colours had 5 μl of 10% sugar solution.

### Floral community spectra

We measured the floral reflectance spectra from 123 plant species within a 2 km radius of the wild hives from which bees were recruited. This distance is well within the known foraging ranges of *A. dorsata* (Young et al., 2021). Reflectance measurements of petals and leaves were taken using a spectrophotometer (Ocean Optics Ocean-HDX UV-vis spectrometer, supplemented by a PX-2 pulsed Xenon light source), with 3–6 replicates per species except for rare species. For each species, one representative spectral curve was used for analysis. To quantify floral contrast against natural backgrounds, which is a key determinant of how bees perceive and detect flowers (Hempel de Ibarra et al., 2015; van der Kooi and Spaethe, 2025), we also measured the background leaf spectra for 83 species and used the median leaf spectrum as a standard green background.

Floral spectra were then modelled in the bee colour hexagon (Chittka, 1992), which incorporates both receptor excitation and chromatic contrast relative to a natural background. This allowed us to examine the distribution of bee-perceived colours within the local plant community and assess whether certain spectral regions are overrepresented in the floral community. Plants were classified as native (*n*=65) or non-native (*n*=58) using the Kew ‘Plants of the World Online’ database (https://powo.science.kew.org) and analysed both separately and jointly. Each species was assigned to one of the six bee-subjective colour categories corresponding to the sectors of the hexagon model (UV, UV–blue, blue, blue–green, green and UV–green), and then further grouped into short-wavelength (UV, UV–blue, blue) and long-wavelength (blue–green, green, UV–green) categories to evaluate broad spectral distribution in the floral community.

### Statistical analyses

#### Behavioural experiments

All statistical analyses were performed in R (version 4.5.1). In all experiments (1–5), the first and total choices made by bees to each colour were obtained by analysing videos frame-by-frame using QuickTime Player (version 10.5). A test of proportions was used to compare the first choices of bees. Wilcoxon tests were performed to examine if total visits indicated a bias for either colour in tested bees. Based on the first choice of a bee (either blue or yellow), a constancy index (CI) was calculated for each individual in each experimental treatment using the sequence of transitions between the two colours. Floral constancy was quantified as the proportion of sequential visits made to the same stimulus colour, following Waser (1986), an approach widely used in studies of pollinator foraging behaviour. The index captures the tendency of bees either to switch between colours or to remain consistent with one colour (Slaa et al., 1998) and was calculated as:
(1)

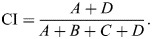


A transition matrix was constructed to categorise transitions such that *A* and *D* represent the frequency of transitions within a colour (‘like’ transitions: blue to blue or yellow to yellow), whereas *B* and *C* represent the frequency of transitions between dissimilar colours (‘unlike’ transitions: blue to yellow or yellow to blue). CI values ranged from 0 to 1, where 0 indicates complete inconsistency, 0.5 indicates random foraging and 1 indicates maximum constancy to one colour.

Additionally, we used a beta regression model to analyse the proportion of total visits to the blue stimulus and the constancy index (CI) across experiments addressing spontaneous constancy under different reward paradigms (in experiments 1–3). The Beta regression was performed using the *betareg* package in R (https://CRAN.R-project.org/package=betareg; Cribari-Neto and Zeileis, 2010). The model was specified as the proportion of visits∼treatment. *Post hoc* and pairwise comparisons were done using the least squares means method with Tukey adjustment by employing the *emmeans* package in R (https://CRAN.R-project.org/package=emmeans; Searle et al., 1980).

#### Community floral spectra

The proportion of flowers that were binned into short-wavelength and long-wavelength categories were compared using a test of proportions to determine if the community was dominated by one of these categories.

## RESULTS

### Spontaneous constancy to colour

**Fig. 2.**
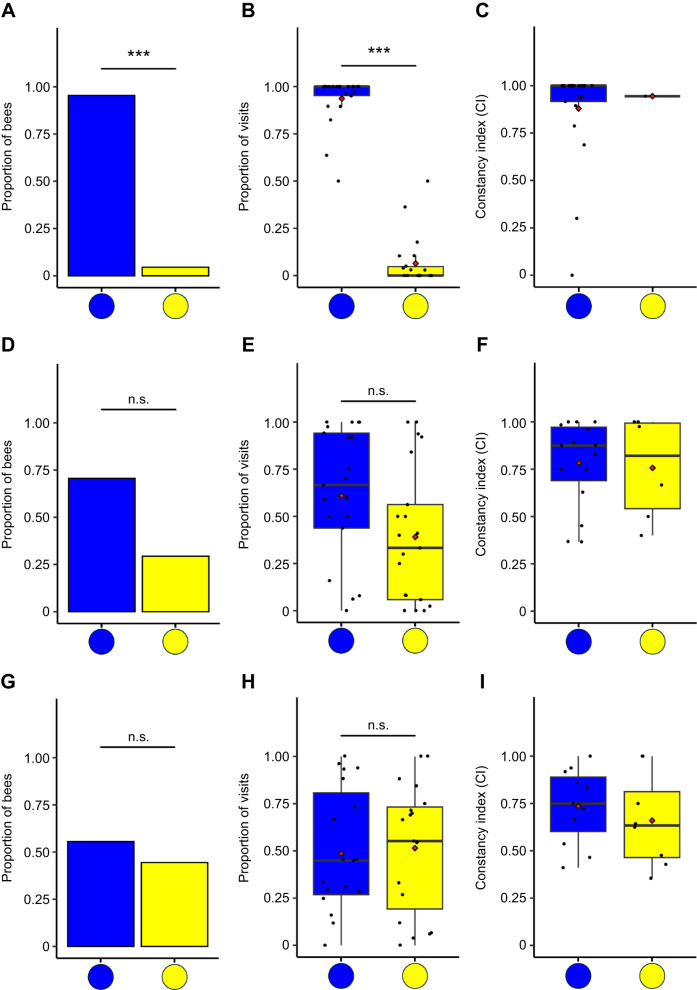
**Training *Apis dorsata* to a UV–grey stimulus with varying levels of sucrose reward.** (A–C) When bees were trained and tested with low concentration sucrose reward (experiment 1), first choices were strongly biased to blue (A), the proportion of total visits to either colour was also biased to blue (B) and the constancy of bees for blue or yellow was similar and high (C). (D–F) When trained to high concentration reward and tested with low concentration reward (experiment 2): first choices were not significantly biased to blue or yellow (D), total choices were also not significantly biased to either colour (E) and constancy was high (F). (G–I) When trained and tested with high concentration reward (experiment 3), first choices were not significantly biased to either colour (G), total choices were also not significantly biased to either blue or yellow (H) and constancy was high (I). The hinges (horizontal bounds of the box) correspond to the interquartile range (IQR), the bold horizontal line corresponds to the median and the whiskers enclose the range of the data. The black points (•) represent data points for corresponding metrics calculated for individual bees in trials and the red point (♦) represents the mean. ***P<0.001.

After training to neutral UV–grey associated with low-concentration reward, bees showed a strong preference for blue in tests, with 21 out of 22 bees choosing blue in their first choice (experiment 1: χ^2^=16.409, d.f.=1, *P*<0.001, *n*=22; Fig. 2A) and when total choices were considered (*v*=231, d.f.=1, *P*<0.001, *n*=22; Fig. 2B) during tests. Constancy index (CI) was also close to one for bees that chose blue as well as for the single bee that chose yellow (Fig. 2C).

Bees that were trained to neutral UV–grey stimuli, containing high-concentration reward (30% w/w sucrose solution) and tested on the blue and yellow array containing low-concentration reward (10% w/w sucrose solution) did not show a significant bias for any colour in their first (experiment 2: χ^2^=3.048, d.f.=1, *P*=0.08, *n*=21; Fig. 2D) or total choices (*v*=121, d.f.=1, *P*=0.3, *n*=21; Fig. 2E). Bees that chose either a blue or yellow stimulus as their first choice (first stimulus a bee landed on during the test) remained consistent to this colour largely, thereby showing high values of constancy (mean±s.d. CI=0.78±0.23, *n*=21; Fig. 2F).

When UV–grey trained bees were both trained and tested with high-concentration reward (30% w/w sucrose solution) they did not show a significant bias for either colour in their first (experiment 3: χ^2^=0.21, d.f.=1, *P*=0.64, *n*=19; Fig. 2G) or total choices (*v*=88, d.f.=1, *P*=0.79, *n*=19; Fig. 2H). Bees that landed on a blue or yellow stimulus as their first choice during tests showed similarly high constancy by remaining largely consistent to the first chosen colour (mean±s.d. CI=0.7±0.22, *n*=19; Fig. 2I).

### Effect of recent foraging experience on constancy

**Fig. 3.**
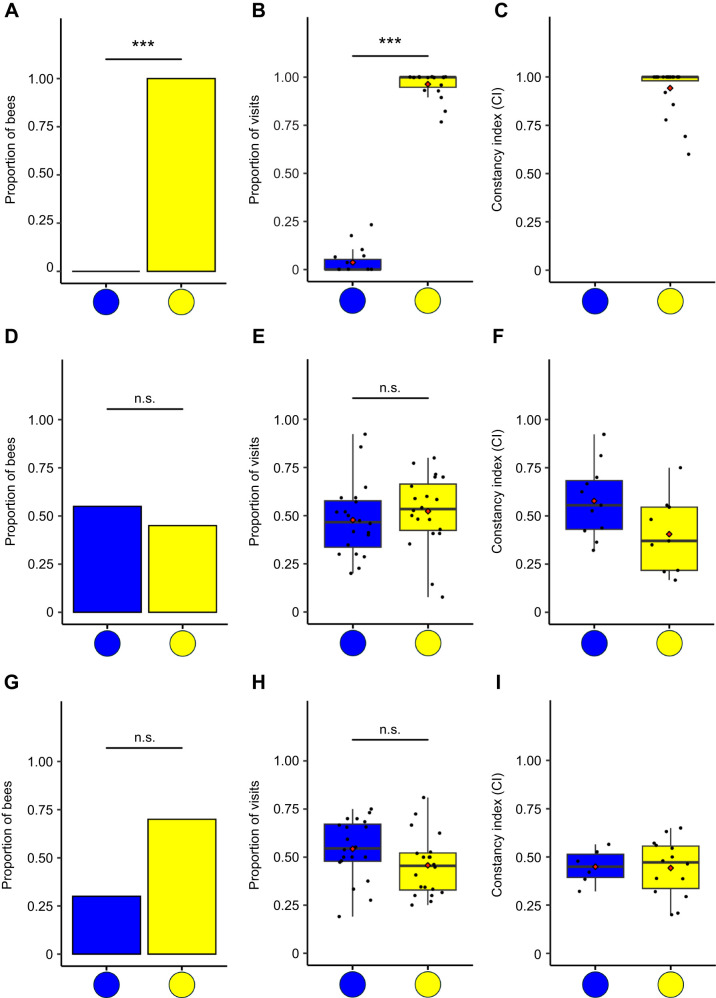
**Effect of recent foraging experience on constancy.** (A–C) When bees were trained with yellow colour (experiment 4), first choices were strongly biased to yellow (A), total choices were also biased to yellow (B) and constancy was high (C). (D–F) When trained to both colours sequentially, with last training to blue, first choices were not biased to blue or yellow (D), total choices were also not biased to blue or yellow (E) and constancy was low (F). (G–I) When trained to both colours sequentially, with last training to yellow (experiment 5), first choices were not biased to blue or yellow (G), total choices were also not biased to blue or yellow (H) and constancy was low (I). The hinges (horizontal bounds of the box) correspond to the interquartile range (IQR), the bold horizontal line corresponds to the median and the whiskers enclose the range of the data. The black points (•) represent data points for corresponding metrics calculated for individual bees in trials and the red point (♦) represents the mean. ***P<0.001.

In experiment 4, when bees were trained to yellow, an unpreferred colour compared with the preferred blue, the first choices of all bees tested were only to yellow (χ^2^=17.05, d.f.=1, *P*<0.001, *n*=20; Fig. 3A) and their total choices were also significantly biased to yellow (*v*=0, d.f.=1, *P*<0.001, *n*=20; Fig. 3C). Consequently, the constancy index was also high (mean±s.d. CI=0.94±0.12, *n*=20; Fig. 3B).

In experiment 5, bees were trained to both blue and yellow in an alternating sequence with one set of bees starting their training sequence with blue and ending with yellow and the other set starting with yellow and ending with blue. Both these groups showed no preference for either colour irrespective of whether they were last trained to blue (χ^2=^0.05, d.f.=1, *P*=0.82, *n*=20; Fig. 3D) or yellow (χ^2^=2.45, d.f.=1, *P*=0.117, *n*=20; Fig. 3G) in their first choices, as well as in total choices when last trained to blue (*v*=113, d.f.=1, *P*=0.47, *n*=20; Fig. 3F) or yellow (*v*=97.5, d.f.=1, *P*=0.32, *n*=20; Fig. 3I). When compared with previous experiments (experiments 1–4), constancy was lower in these sequentially trained bees regardless of their last training i.e. last trained to blue (mean±s.d. CI=0.37±0.15, *n*=20; Fig. 3E) or last trained to yellow (mean±s.d. CI=0.31±0.11, *n*=20; Fig. 3H), suggesting a random foraging pattern.

### Comparison of bee preferences and constancy across experiments

Using a beta regression model, we examined the proportion of total visits to the blue stimuli and constancy across experiments 1–3. We tested whether there were significant differences in constancy and colour preference between bees trained to UV–grey under various reward paradigms. Preference to blue was significantly reduced when the bees experienced higher quality reward in training alone or both during training and test [beta regression (formula=proportion of visits∼treatment) and pairwise comparison of marginal means with Tukey adjustment, *P*<0.001; Fig. S4A, Table S2]. Constancy was high (>0.7) in all three experiments [beta regression (formula=constancy index∼treatment) and pairwise comparison of marginal means with Tukey adjustment, *P*<0.001; Fig. S4B; Table S3].

#### Community flower spectra

**Fig. 4.**
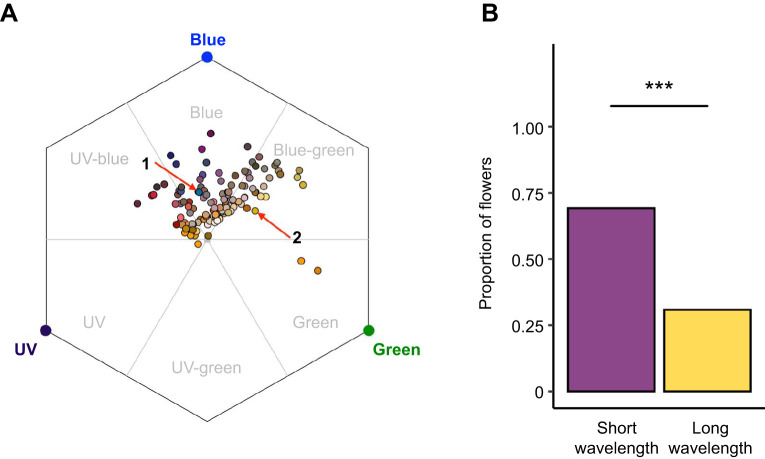
**Community floral spectra in bee-subjective hexagonal colour space, categorised into UV, UV–blue, blue, blue–green, green and UV–green sectors.** (A) A total of 123 flowers in the community were modelled with the background being leaf green. The circles represent the colour loci flowers in the community. The red arrows indicate the blue (labelled 1) and yellow (labelled 2) stimuli used in the experiments. (B) Flowers were categorised into long or short wavelength based on the hexagon sector in which the colour loci of the flowers are located. ***P<0.001.

When the floral reflectance spectra was modelled in the bee hexagonal colour space with a green leaf background (Fig. 4A), the community spectra was found to be significantly shifted toward short wavelengths regardless of whether we considered all flowers (test of proportions, χ^2^=18.732, d.f.=1, *P*<0.001; Fig. 4B), only native species (test of proportions, χ^2^=13.846, d.f.=1, *P*<0.001; Fig. S5A,B) or only non-native species (test of proportions, χ^2^=4.983, d.f.=1, *P*=0.026;  Fig. S5C,D).

## DISCUSSION

We examined how reward concentration modulates spontaneous colour preferences and constancy in the Asian giant honeybee *A. dorsata*. Despite extensive work on pollinator learning, the interaction between reward quality and spontaneous sensory biases remains poorly understood (van der Kooi et al., 2023), especially in tropical bees. We show that reward concentration directly shapes both spontaneous colour preferences and floral constancy, a relationship not previously demonstrated in bees. Consistent with predictions about the interaction between innate biases and learning, we found that spontaneous preferences can be overridden through experience, resulting in strong preferences and high constancy for colours that differ from the spontaneously preferred blue. In contrast, recent colour experience promotes more exploratory foraging behaviour, leading to reduced constancy, highlighting the dynamic nature of foraging decisions under natural conditions. Finally, the observed preference for blue is broadly concordant with the dominant floral colours in the local environment, suggesting a potential link between pollinator biases and community-level floral signals.

Following training to a neutral UV–grey stimulus, bees showed a strong preference for and high constancy to blue when provided with low sucrose concentrations during both training and testing. This suggests that when rewards are low and learned associations weak, bees rely more heavily on their spontaneous colour biases. In support of this interpretation, bees continued to visit the blue stimuli even after rewards were depleted, indicating a preference for blue in *A. dorsata* that is likely to be spontaneous. As mentioned earlier, our experiments were conducted on wild colonies with unknown foraging histories, hence we exercise caution in confirming that this reflects a return to an innate preference. Therefore, we consider it a spontaneous preference without invoking the assumption of a hardwired innate response. The weakening of the blue bias in subsequent experiments, in which bees were exposed to the less preferred yellow, or were sequentially exposed to both blue and yellow, suggests that UV–grey training probably reset prior associations, leading to a reversion to spontaneous preference, as reported in other bee species (Balamurali et al., 2018; Gumbert, 2000).

Some species of honeybees, bumblebees, stingless bees and butterflies exhibit innate preferences for short-wavelength colours (Arikawa et al., 2021; Giurfa et al., 1995; Gumbert, 2000; Kinoshita et al., 2017; Morawetz et al., 2013; Nicholls et al., 2015; Raine and Chittka, 2005a; Yoshida et al., 2015). Bumblebees, for example, revert to their innate blue colour preference when trained to a neutral stimulus (Giurfa et al., 1995; Gumbert, 2000). In contrast, the sympatric eastern honeybee *A. cerana*, exhibits a long-wavelength bias in naïve foragers, as well as in experienced foragers after prolonged enclosure (Balamurali et al., 2018). While this may reflect true species-level differences, variation in experimental design between these studies limits direct comparisons. Our findings add to the growing evidence for interspecific diversity in pollinator colour preferences.

We show that the blue bias depends on reward concentration, being strongest at low sucrose concentrations and weakening at higher concentrations. We propose that reward concentration during UV–grey training modulates arousal in bees. Low rewards favour reliance on innate preferences, whereas high rewards increase arousal and promote exploration, especially when paired with a neutral stimulus like UV–grey that may reset prior associations (Balamurali et al., 2018; Gumbert, 2000). Bees make adaptive, energy-based decisions (Grüter et al., 2011) and higher reward concentration is a rapidly assessed, neurologically salient cue that facilitates learning (Cnaani et al., 2006). Because bees can learn multiple colour–reward associations (Muth et al., 2015) and learn faster at higher reward concentrations, individuals in our high-reward treatments probably sampled both colours during tests. As both offered identical rewards, this reduced floral constancy. Consistent with this, higher rewards enhance attention and decision accuracy (Nityananda and Chittka, 2021), whereas constancy reflects trade-offs between exploration and floral distinctiveness (Hernández et al., 2025). Mechanistically, high rewards trigger octopamine and dopamine release in the brain, promoting appetitive behaviour (Perry and Barron, 2013) and lower the response thresholds to novel stimuli (Scheiner et al., 1999). Accordingly, bees under high reward conditions, distributed visits across both colours to maximize the energy intake in what was likely to be a heightened state of arousal. By contrast, the low reward group relied on spontaneous preferences and chose blue stimuli without exploring the yellow stimuli. Importantly, unlike previous studies on learned associations, our results show that spontaneous preferences and constancy are themselves modulated by reward exposure.

Brief experience with one or both colours reversed the spontaneous preference, indicating rapid learning and exploration. While multisensory modulation of colour preferences by olfactory cues is known in butterflies (Anaswara et al., 2025; Yoshida et al., 2015) and multisensory integration has been shown to improve flower detection in bumblebees (Kunze, 2001), the modulation of spontaneous colour preference by reward quality has not been previously demonstrated. Given that reward processing is mediated by biogenic amines in insect brains (Perry and Barron, 2013), spontaneous colour preferences are likely to be flexibly regulated by reward value. Previous studies that used similarly high sucrose concentrations (30% w/v or w/w) in *A. mellifera* and *A. cerana* reported different patterns. In *A. mellifera*, high reward concentrations produce strong individual constancy without a consistent population-level colour bias, whereas lower concentrations reduce constancy without inducing colour preference (Grüter et al., 2011; Grüter and Ratnieks, 2011; Wells et al., 1992). In *A. cerana*, foragers often show low constancy and visit multiple colours (Wells and Rathore, 1994). These differences suggest that reward expectation shapes exploitation and exploration when bees encounter unfamiliar stimuli (Gil, 2010; Solvi et al., 2016). Our results extend this framework by showing for the first time that reward quality can directly modulate spontaneous sensory biases.

As expected, the spontaneous preference was flexible and readily modified by learning, which is consistent with previous studies (e.g. Nicholls et al., 2015). Bees trained to the less preferred yellow, shifted their colour preference and exhibited high constancy. Even brief experience is sufficient for colour learning, especially for stimuli that contrast strongly with the background (Balamurali et al., 2018; Menzel, 1967; Niggebrügge et al., 2009). Bees also learned two colours simultaneously and used this information when making foraging choices, which is consistent with findings in bumblebees (Muth et al., 2015, 2017). Previous studies using similar arrays have attempted to train bees to two colours but introduced longer temporal gaps between the training colours, which may bias the bees towards the most recently trained colour (Hill et al., 1997). In contrast, we show that when trained to both colours sequentially and in quick succession, bees visit both colour stimuli extensively and frequently switch between them. This resulted in a more random foraging pattern and low constancy, with no detectable effect of the last trained colour on test outcomes. Constraints on the memory of bees, especially short-term memory, have been proposed as a mechanism of constancy (Chittka et al., 1999; Gegear and Laverty, 2001, 2005; Hill et al., 1997, 2001; Waser, 1986). However, our results, along with recent studies showing that bees can flexibly learn and use multiple floral cues even over short timescales (Ings et al., 2009; Muth et al., 2015; Nicholls et al., 2015; Raine and Chittka, 2007a), suggest that memory constraints alone may not fully explain patterns of constancy, at least when floral traits are simple, as in our experimental stimuli.

Floral colours in the study community were strongly biased towards short-wavelength hues. This pattern remained consistent whether native and non-native species were analysed separately or together. Our experiments suggest that *A. dorsata* exhibits a spontaneous preference for blue. However, more extensive sampling of the region's floral diversity, along with assessments of the spontaneous colour preferences of other dominant pollinators, will be necessary to fully understand whether the observed correspondence between floral spectra and pollinator biases reflects pollinator-driven selection pressures. Community floral colour assemblages are known to be shaped by the dominant pollinators in a habitat (Chittka and Menzel, 1992; Ishii et al., 2019; Lunau and Maier, 1995; Reverté et al., 2016), with the most rewarding floral colours sometimes reflecting pollinator biases (Chittka et al., 2004; Raine and Chittka, 2007b; Streinzer et al., 2021). The high prevalence of bee-subjective short-wavelength floral colour (Chittka, 1992; Fig. 4A,B) in our community is consistent with patterns documented in some other regions, where insect pollination dominates (Arnold et al., 2009; Chittka et al., 2004; Dyer et al., 2012, 2021; Ortiz et al., 2021; Shrestha et al., 2014, 2019).

Innate preferences may facilitate initial flower detection and are assumed to have evolved to guide pollinators toward the most rewarding floral types (Giurfa et al., 1995; Lunau and Maier, 1995; Raine and Chittka, 2007b), although not always (Shrestha et al., 2020). Alternative explanations include resource partitioning (Balamurali et al., 2018) and the enhanced detectability of short-wavelength signals against natural backgrounds (Bukovac et al., 2017). Some studies suggest that blue flowers are detected and foraged on more efficiently than other colour morphs of the same species (Dyer et al., 2007; Waser and Price, 1985). Taken together, the predominance of blue, UV–blue and blue–green flowers in the study site may reflect pollinator-mediated selection on floral colour (Finnell and Koski, 2021; van der Kooi et al., 2016). Interestingly, naïve *A. cerana* in the same region show a spontaneous bias for long wavelength colours and naïve stingless bees, *T. iridipennis* show bias for blue colour (Balamurali et al., 2018). These species-specific differences may reflect resource partitioning between sympatric bees. However, testing truly innate preferences in *A. dorsata* is extremely challenging because of its open nesting habit and the uncontrollable foraging history of individuals. We controlled the short-term experience by extensively training bees on neutral UV–grey stimuli and by keeping the interval between training bouts, and between the last training and test under 2 min. Both spontaneous and learned preferences shape pollinator foraging (Kinoshita et al., 2017; Raine and Chittka, 2005a). Although spontaneous preferences can initially reduce foraging efficiency, bees rapidly learn and adjust their behaviour (Menzel, 1985; Morawetz et al., 2013; Muth et al., 2015; Srinivasan, 2010). Moreover, constancy is influenced by multiple interacting factors, including sensory cues, floral signals, energetic constraints on learning and memory, social information and the spatial distribution of flowers (Abts and Dunlap, 2022; Baracchi et al., 2018; Bruninga-Socolar et al., 2022; Gegear and Laverty, 2005; Takagi and Ohashi, 2025). Thus, constancy remains an adaptive strategy even in the presence of spontaneous biases.

Finally, our artificial flowers were deliberately kept simple, differing only in colour to enable controlled comparisons across studies (Gegear and Laverty, 2004; Grant, 1950; Grüter et al., 2011; Grüter and Ratnieks, 2011; Hill et al., 1997, 2001; Wells et al., 1992; Wells and Rathore, 1994; Wells and Wells, 1983). However, natural flowers display complex, multimodal signals. Bee behaviour and constancy can change when flowers vary in morphological complexity, number of morphs or spatial arrangement (Takagi and Ohashi, 2025). Bees also integrate information from multiple modalities which include colour, odour, shape and size, while making foraging decisions (e.g. Gegear and Laverty, 2005; Slaa et al., 1998, 2003). Incorporating such complexity will be essential for understanding constancy in natural settings. Notably, *A. dorsata* differs behaviourally from both *A. mellifera* and *A. cerana* under similar test conditions (Wells and Rathore, 1994), underscoring the importance of more species-specific studies (Raine and Chittka, 2005b; Slaa et al., 1998; Wells and Rathore, 1994). More broadly, our results demonstrate that spontaneous sensory biases are not fixed, but are dynamically modulated by reward quality, with consequences for foraging behaviour and potentially for the structure of plant–pollinator interactions.

## Supplementary Material

10.1242/jexbio.252021_sup1 Supplementary information
