# Supplementary material for: Colour preference and foraging constancy in the Asian giant honeybee Apis dorsata
Source: J Exp Biol. 2026 Jun 4;229(11):jeb252021. doi: 10.1242/jeb.252021 (PMC13286372; doi:10.1242/jeb.252021)
Supplement: Supplementary information [file jexbio-229-252021-s1.pdf]

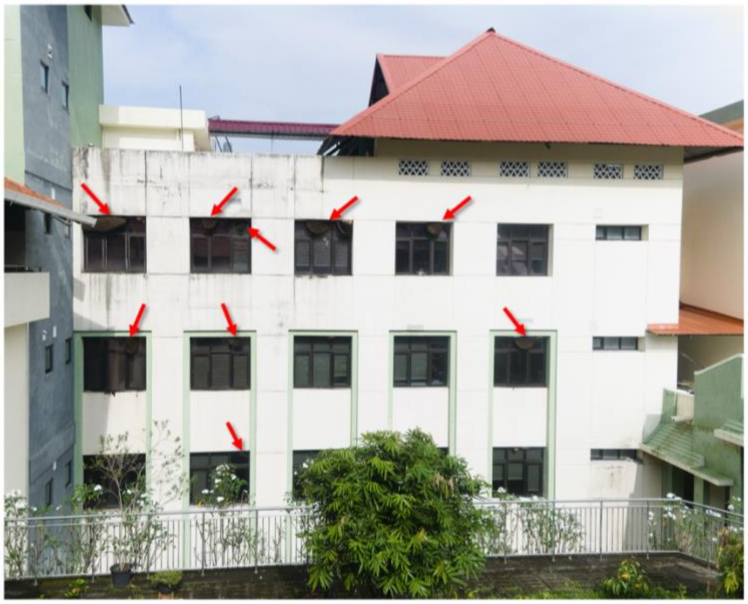

Fig. S1. *A. dorsata* colonies on the window ledges of the Biological Sciences Building, IISER Thiruvananthapuram. The red arrows point to individual colonies from which bees were recruited to the roof terrace for experiments.

Journal of Experimental Biology • Supplementary information

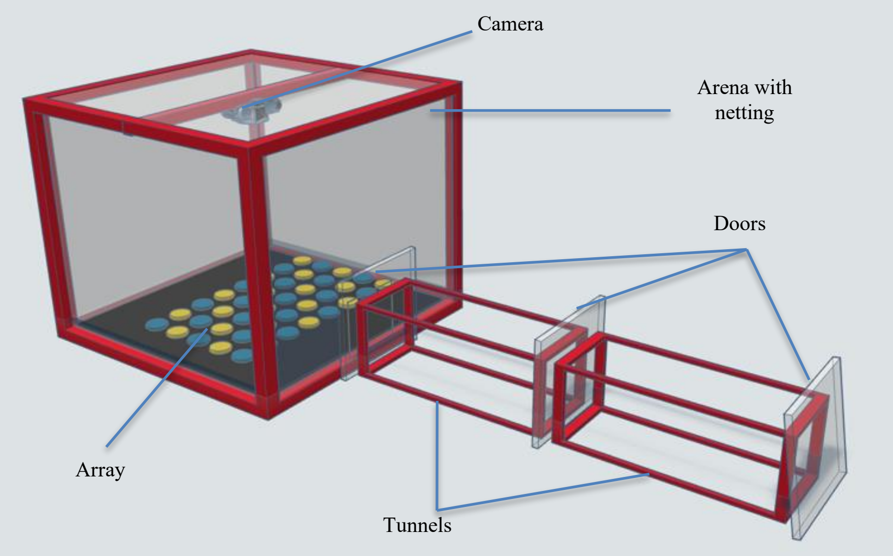

Fig. S2. Experimental arena with netting on all sides and a clear plexiglass tunnel leading to the interior of the arena. The three doors in the tunnel help to control the number of bees entering. A camera mounted on a cross bar on the roof was used to record the choices of bees in tests.

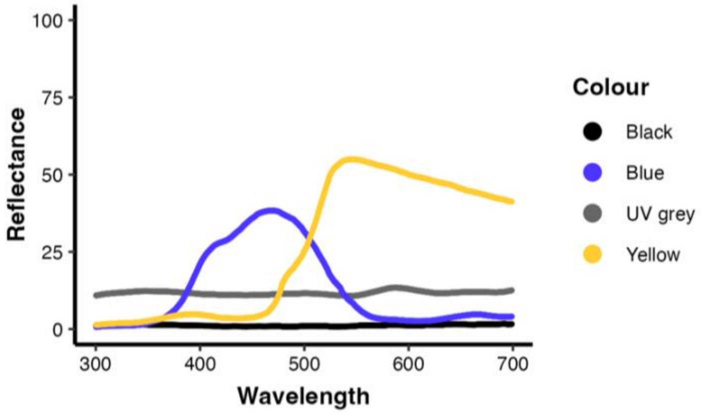

Fig. S3. Spectral reflectance of the blue and yellow stimuli, the neutral UV-grey stimulus and the black background against which the colour stimuli were presented in the different experiments.

Journal of Experimental Biology • Supplementary information

A

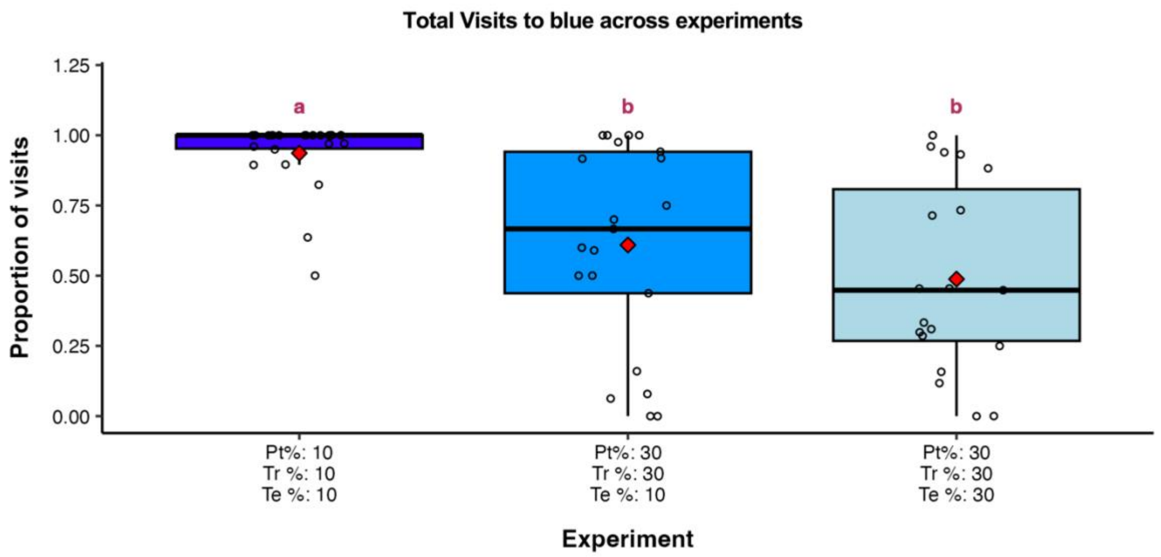

B

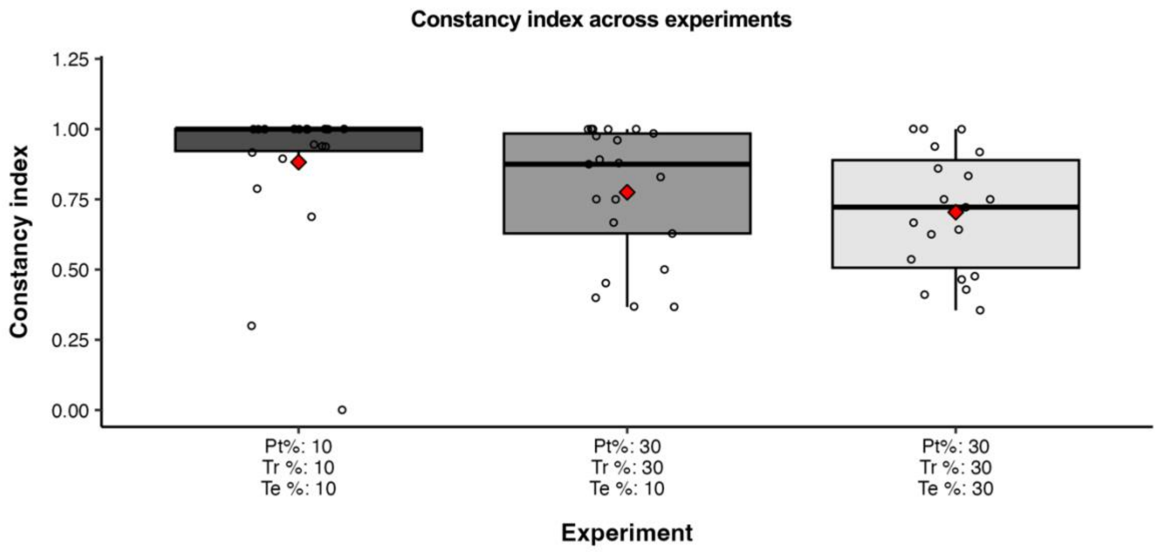

Fig. S4. A) Comparison of proportion of visits to blue stimuli in tests and B) constancy index across *experiments 1,2 and 3* (*left to right*). Pt% stands for pre-training reward concentration, Tr% stands for training reward concentration and Te% stands for test reward concentration. The *hinges* (horizontal bounds of the box) correspond to the interquartile range (IQR), the *bold horizontal line* corresponds to the median and the *whiskers* enclose the range of the data. The open circles (o) represent data points for corresponding metrics calculated for individual bees in the trials and the red point (◆) represents the mean. The letters above the box plots are compact letter display with means not sharing any letter being significantly different using pairwise comparisons employing least squares means method with Tukey adjustment at 5% level of significance.

Journal of Experimental Biology • Supplementary information

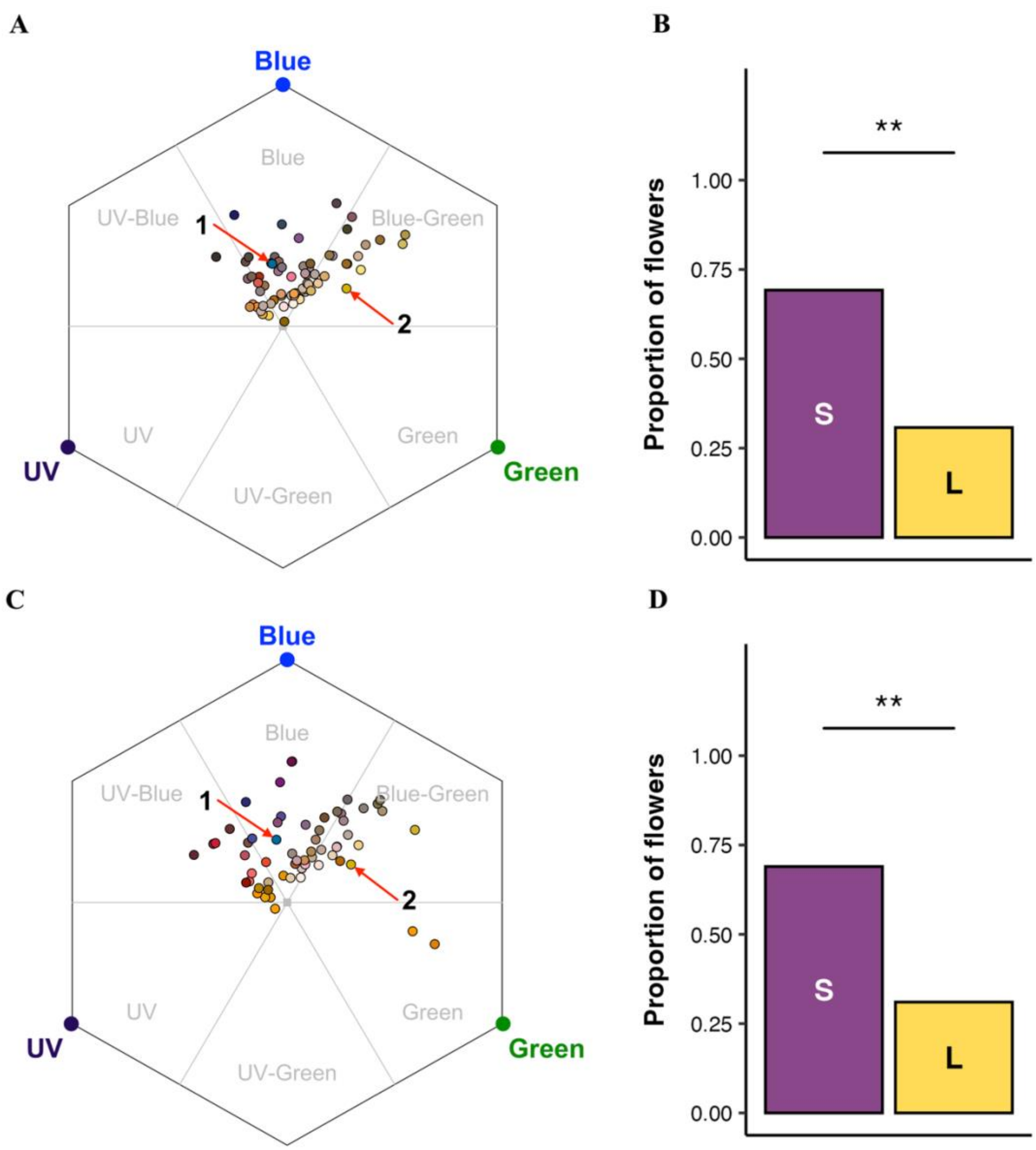

Fig. S5. Community floral spectra in bee-subjective hexagonal colour space, categorised into long and short wavelength A) Native flowers (n=65) modelled in the bee hexagonal colour space with the background being a leaf green. B) The native flowers (n=65) categorised into short and long wavelength colours (labelled "S" and "L" inside the bars) based on the hexagon sector. C) Exotic flowers (n=58) modelled in the bee hexagonal colour space with the background being a leaf green. D) Exotic flowers (n=58) categorised into short and long wavelength colours (labelled "S" and "L" inside the bars) based on hexagon sector. The circles (o) represent the colour loci flowers in the community. Flowers that occur in the UV, UV-blue and blue sectors were classified as short wavelength flowers and those that occur in blue-green, green and UV-green sectors were classified as long wavelength colours. The red arrows point to the blue (labelled 1) and yellow (labelled 2) stimuli used in the experiments. A significant proportion of flowers belonged to the short wavelength category. ** depicts significance at $p <$ 0.01.

Table S1. Colour contrast of the stimuli used in the experiment modelled with black background in the bee-specific hexagonal colour space. Values **x** and **y** are the Cartesian coordinates of a colour locus within the hexagonal colour space. **r.vec** represents the r-vector, calculated as the Euclidean distance from the achromatic centre to the colour locus.

	Stimuli	x	y	r.vec
1	Blue	0.103	0.070	0.125
2	Yellow	0.273	0.025	0.274
3	UV-grey	0.018	-0.001	0.018

Table S2. Pairwise comparison of proportion of total visits to the blue colour stimuli across *experiments 1, 2 and 3* with beta regression (formula = frequency of visits ~ treatment) using marginal means method and Tukey adjustment.

Quantile residuals:

Min	1Q	Median	3Q	Max
-2.455	-0.340	-0.015	0.603	2.149

Log-likelihood: 69.71 on 4 d.f.

Pseudo R-squared: 0.322

AIC: -131.418

Coefficients:

	component	term	estimate	SE	statistic	p value
1	mean	*Experiment 1* (Intercept)	1.542	0.274	5.620	1.91e-08
2	mean	*Experiment 2*<br>Training concentration: 30%<br>Test concentration: 10%	-1.129	0.376	-3.000	0.003
3	mean	*Experiment 3*<br>Training concentration: 30%<br>Test concentration: 30%	-1.609	0.402	-4.001	6.31e-05

Journal of Experimental Biology • Supplementary information

Emmeans:

	experiment	estimate	SE	95% CI (Lower)	95% CI (Upper)
1	*Experiment 1*<br>Training concentration: 10%<br>Test concentration: 10%	0.824	0.040	0.746	0.902
2	*Experiment 2*<br>Training concentration: 30%<br>Test concentration: 10%	0.602	0.066	0.472	0.732
3	*Experiment 3*<br>Training concentration: 30%<br>Test concentration: 30%	0.483	0.073	0.341	0.626

Pairwise contrast:

	contrast	estimate	SE	Z-ratio	p value
1	*Experiment 1*<br>Training concentration: 10%<br>Test concentration: 10%<br>vs<br>*Experiment 2*<br>Training concentration: 30%<br>Test concentration: 10%	0.222	0.075	2.955	0.009
2	*Experiment 1*<br>Training concentration: 10%<br>Test concentration: 10%<br>vs<br>*Experiment 3*<br>Training concentration: 30%<br>Test concentration: 30%	0.340	0.083	4.090	0.000
3	*Experiment 2*<br>Training concentration: 30%<br>Test concentration: 10%<br>vs<br>*Experiment 3*<br>Training concentration: 30%<br>Test concentration: 30%	0.119	0.099	1.201	0.453

Table S3. Pairwise comparison of constancy index across experiments 1,2 and 3 with beta regression (formula = CI ~ treatment) using marginal means and Tukey adjustment.

Quantile residuals:

Min	1Q	Median	3Q	Max
-4.062	-0.589	-0.078	0.772	1.703

Log-likelihood: 73.98 on 4 d.f.

Pseudo R-squared: 0.124

AIC: -139.968

Coefficients:

	component	term	estimate	SE	statistic	p value
1	mean	*Experiment 1* (Intercept)	1.727	0.272	6.344	2.24e-
2	mean	*Experiment 2*<br>Training concentration: 30%<br>Test concentration: 10%	-0.527	0.345	-1.527	0.127
3	mean	*Experiment 3*<br>Training concentration: 30%<br>Test concentration: 30%	-0.872	0.360	-2.422	0.015

Emmeans:

	experiment	estimate	SE	95% CI (Lower)	95% CI (Upper)
1	*Experiment 1*<br>Training concentration: 10%<br>Test concentration: 10%	0.849	0.035	0.781	0.917
2	*Experiment 2*<br>Training concentration: 30%<br>Test concentration: 10%	0.769	0.048	0.674	0.863
3	*Experiment 3*<br>Training concentration: 30%<br>Test concentration: 30%	0.702	0.058	0.588	0.815

Pairwise contrast:

	contrast	estimate	SE	Z-ratio	p value
1	*Experiment 1*<br>Training concentration: 10%<br>Test concentration: 10%<br>vs<br>*Experiment 2*<br>Training concentration: 30%<br>Test concentration: 10%	0.080	0.054	1.498	0.292
2	*Experiment 1*<br>Training concentration: 10%<br>Test concentration: 10%<br>vs<br>*Experiment 3*<br>Training concentration: 30%<br>Test concentration: 30%	0.147	0.063	2.327	0.052
3	*Experiment 2*<br>Training concentration: 30%<br>Test concentration: 10%<br>vs<br>*Experiment 3*<br>Training concentration: 30%<br>Test concentration: 30%	0.067	0.071	0.942	0.614

Journal of Experimental Biology • Supplementary information
